# Internal translation of truncated protein isoforms throughout the connexin gene family

**DOI:** 10.1016/j.gendis.2025.101572

**Published:** 2025-02-25

**Authors:** María José Muñoz, Alejandro Cabrera-Andrade, Ane Gutierrez-Aguirregabiria, Ipek Ilgin Gönenc, Santiago Ramón y Cajal, Trond Aasen

**Affiliations:** a Patologia Molecular Translacional, Vall d’Hebron Institut de Recerca (VHIR), Vall d’Hebron Hospital Universitari, Vall d’Hebron Barcelona Hospital Campus, Passeig Vall d’Hebron 119-129, Barcelona 08035, Spain; b Grupo de Bio-Quimioinformática, Universidad de Las Américas, Quito 170124, Ecuador; c Equipo DeustoMED- Departamento de Medicina, Facultad de Ciencias de la Salud- Universidad de Deusto, Av de las Universidades 24, Bilbao 48007, Spain; d Center for Chromosome Stability, Department of Cellular and Molecular Medicine, University of Copenhagen, Copenhagen N 2200, Denmark; e CIBER de Cáncer (CIBERONC), Instituto de Salud Carlos III, Avenida de Monforte de Lemos 3-5, Madrid 28029, Spain; f Universitat Autònoma de Barcelona, Bellaterra 08193, Spain

The connexin family of mammalian gap junction proteins provides intercellular communication channels critical for development and tissue function. Mutations in connexins are associated with numerous diseases including deafness, skin diseases, cataracts, cardiac diseases, neurological diseases, cancer, and complex syndromic disorders. Connexin 43 (Cx43, encoded by *GJA1*) is the most widely expressed and studied member. We and others have shown that, through a poorly understood mechanism of direct internal translation initiation, the single coding exon of *GJA1* generates various N-terminally truncated Cx43 forms, notably the 20 kDa (k) form GJA1-20k.^1^^,^^2^ Numerous functions have subsequently been ascribed to these truncated forms, providing critical insight into non-canonical *GJA1* functions.^3^

Here, we expand on these observations within the connexin gene family. Cap-dependent ribosomal scanning is required to translate truncated full-length (FL) but also truncated Cx43 isoforms^2^ making us hypothesize that, despite a strong Kozak context, leaky ribosomal scanning may regulate the synthesis of truncated Cx43 forms. Weakening the Kozak region reduced FL Cx43 synthesis, whereas removing the consensus ATG start codon M1 (ΔM1) or introducing an out-of-frame (OOF) stop codon abolished FL Cx43. Yet, the expression of truncated forms of Cx43 was maintained or increased (Fig. 1A–C) with noteworthy cell line-specific differences in presumed phosphorylation patterns of truncated isoforms, which curiously seems influenced by the presence of FL Cx43.

**Figure 1:**
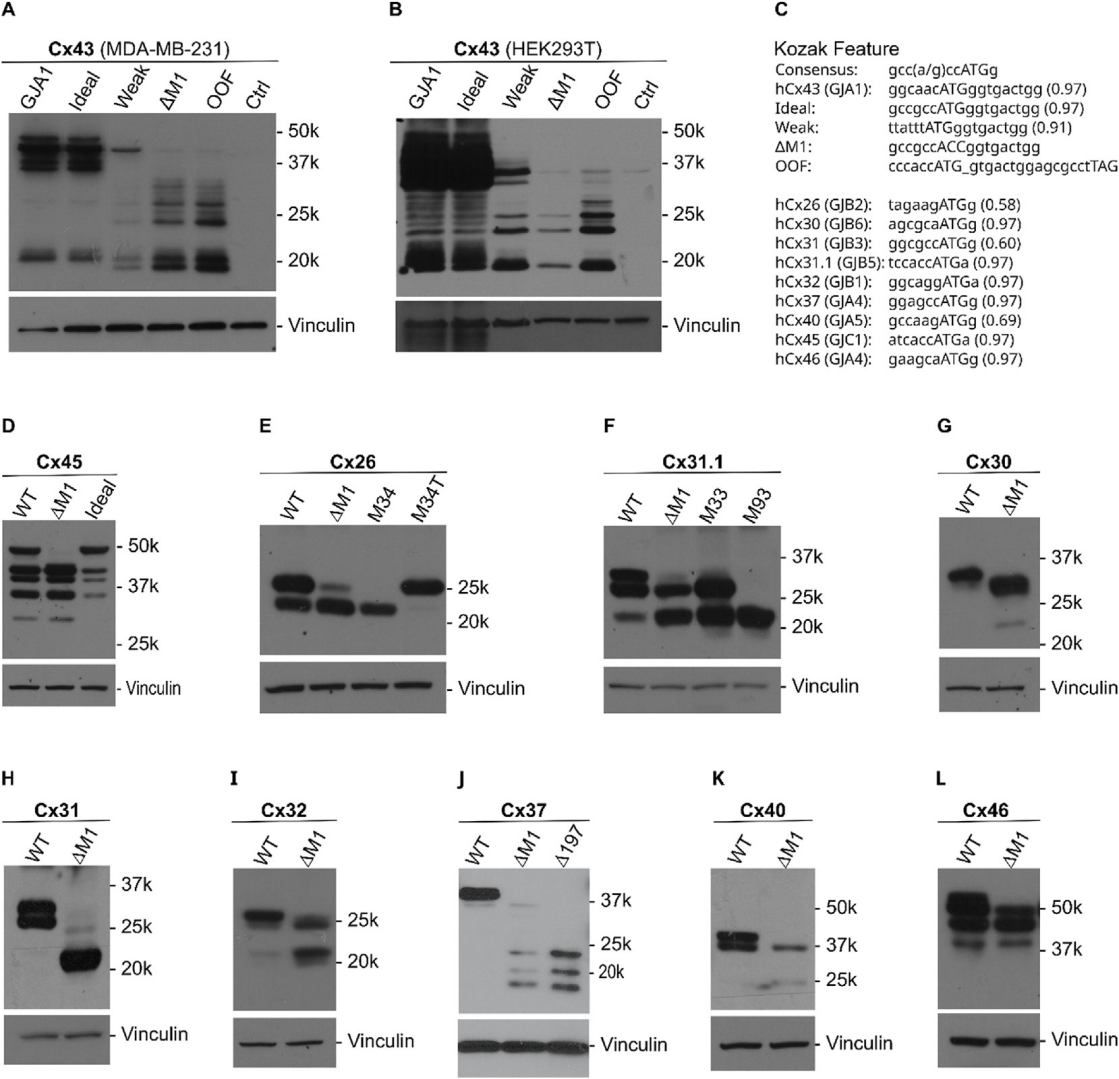
Internal translation of truncated protein isoforms within the connexin gene family. **(A, B)** Examples of Cx43 expression together with truncated isoforms in two different cell lines. Reducing translation of full-length (FL) Cx43, or halting it altogether (ΔM1 deletion of ATG initiation site or introducing an OOF mutation), maintained or increased the expression of truncated isoforms. Note the different phosphorylation patterns occurring in the two cell lines, which seem influenced by the presence or absence of FL protein. **(C)** Details of Cx43 (*GJA1*) mutations and Kozak regions for each gene with their predicted translation initiation score (see Table S1). **(D)** Cx45 translated FL as well as truncated versions that were maintained when deleting FL protein. Truncated isoform intensity was reduced when optimizing the Kozak region. **(E)** Cx26 yielded two intense bands of approximately 26 and 23 k. The 23 k band was maintained in the ΔM1 version. A faint band was still observed around 25 k. **(F)** Cx31.1-myc displayed two intense bands (around 26–32 k) and another band around 21 k. ΔM1 eliminated the slowest migrating 32 k band (presumably Cx31.1). The 26 k band was maintained and the 21 k band increased. **(G)** Cx30 only displayed a faint extra band migrating slightly faster than the main band around 27 k, correlating to the most predicted (Table S1) translated isoform (27 k), which was significantly enhanced in the ΔM1 version (with an additional band around 20 k, corresponding to the third predicted form). **(H)** Cx31 displayed two similarly intense bands around 26–32 k (FL + a second band not predicted). However, both bands disappeared in the ΔM1 version, suggesting that either post-translational modification or cleavage is responsible for these two distinct bands. An additional smaller band around 22 k was observed in the ΔM1 setting, which may be an artifact. Indeed, the only truncated protein predicted *in silico* is a 12 k protein, a size fraction we have not assayed in detail. **(I)** Cx32 is known to run in the range of 26–28 k and Cx32-myc produced a major band at around 28 k with faint bands at 25 and 22 k. The *in silico* analysis predicted forms that may correspond to this, at 32 k (FL Cx32, 1st), 28 k (2nd), and 22 k (3rd). As expected, the 28 k presumed FL Cx32 band disappeared in the ΔM1 construct, and the 28 and 22 k bands were more highly expressed, suggesting a mechanism of internal translation and not Cx32 cleavage products. Faint bands surrounding these two bands may be additional products or post-translational modifications such as phosphorylation. **(J)** For Cx37-myc, we mainly observed a 37 k FL protein with a very faint band at around 36k which was maintained in the ΔM1 variant concurrent with the appearance of the bands of around 23, 21, and 19 k. The top three bands truncated forms suggested *in silico* were 17, 22, and 20 k. Deleting bp 1–197 (Δ197) eliminated the 36k band but maintained the more intense three bands as in the ΔM1 version. RegRNA 2.0 analysis predicted a TTG initiation site at bp 28–30. **(K)** Cx40 manifested two very similar bands. The first corresponds to the FL protein at 40 k; the second one, around 37 k, was not predicted by ATGgr (Table S1) but could be derived from a GTG-based initiation codon at bp 67 as predicted by RNA Reg 2.0. **(L)** Cx46 produced four bands in the 45–55 k region. Only the upper band (presumed FL) disappeared with ΔM1. The other truncated isoforms in this region were not predicted *in silico*, which could point to a poor understanding of translation initiation (particularly the use of alternative translation initiation codons such as CTG and GTG, but could also point to technical artifacts and the need for continuous research in this field).

We used the *in silico* online tool ATGpr (https://atgpr.dbcls.jp/) to predict the five most probable translated proteins (Table S1). As expected, FL (M1) initiation sites scored highest. From 22 potential *GJA1* ATGs in the coding sequence, the second predicted isoform, starting at basepair (bp) 636 (M213, ATG number 15, calculated molecular weight 18.5 k), corresponds to GJA1-20k, the most widely expressed and previously verified isoform^1^^,^^2^ (Fig. 1A, B). Subsequently, translation initiation was predicted at bp 373 (M125, calculated molecular weight 18.5 k) and bp 841 (M281, 11.3 k), corresponding to the previously verified isoforms GJA1-28k and GJA1-11k, respectively.^1^ Finally, a 9 k form was predicted from another open reading frame unrelated to Cx43.

To expand this concept to other connexins, we cloned another nine family members, Cx32 (*GJB2*), Cx30 (*GJB6*), Cx31 (*GJB3*), Cx31.1 (*GJB5*), Cx32 (*GJB1*), Cx37 (*GJA4*), Cx40 (*GJA5*), Cx45 (*GJC1*), and Cx46 (*GJA4*)), including the 6 bp upstream Kozak region. Translation of truncated isoforms was predicted *in silico* in all cases (Table S1). To verify the potential role of leaky ribosomal scanning and the caveat that truncated isoforms are cleavage products of FL connexins, we generated ΔM1 versions in which the ATG initiation site (M1) for the FL protein was deleted. We added a C-terminal myc-tag (1.2 k) to facilitate easy and clean immunoblots. As predicted *in silico* (Table S1), we detected N-terminally truncated isoforms experimentally (Fig. 1). Several connexins caught our attention and were analyzed further.

Cx45 is similar to Cx43 in size but contains a weaker Kozak context (atcaccATGa) (Fig. 1C). Upon transfecting 293T cells to express Cx45-myc, we readily detected a presumptive Cx45 band just under 50 k in size plus additional intense bands of lower molecular weight of around 45, 40, 35, and 30 k (Fig. 1D). Only the 35 k band likely corresponds to predicted isoforms (18, 34 and 16 k), although the 40 k band may be a modified (*e.g.*, phosphorylated) form of the 35 k protein. Indeed, the observed migratory sizes of connexins are notoriously difficult to predict, and any assumptive predictions must ultimately be verified experimentally (as shown for Cx43 previously^1^^,^^2^). Of note, the larger truncated Cx45 isoforms may translate from alternative translation initiation codons, for which the RegRNA 2.0 software^4^ identified two GTG start sites (bp 73 and 167) within the coding sequence. To determine the correct FL Cx45 band (or bands depending on phosphorylation status), and to exclude that the smaller isoforms were Cx45 degradation or cleavage products, we deleted the FL Cx45 start codon (ΔM1). As seen in Figure 1C, ΔM1 Cx45 produces all the observed truncated isoforms (but not the heaviest FL form, as expected). We then modified the Kozak region into a consensus (gccaccATGg) sequence. This substantially reduced the presence of the truncated isoforms (Fig. 1D), further suggesting leaky ribosomal scanning and not alternative splicing regulate the synthesis of truncated Cx45 isoforms.

Cx26-myc (with a poorly predicted M1 score of 0.58, Fig. 1C) yielded two intense bands of approximately 26 and 23 k (Fig. 1E). The 23 k version was maintained in the ΔM1 version. Curiously, a faint band was still observed around 25 k, which may derive from a non-canonical GGT translation initiation site at bp 37–39 as predicted by RegRNA 2.0 in the absence of ΔM1. We focused on the 23 k band, which coincides with the top predicted isoform *in silico*, initiating at M34 (22.5 k). This caught our attention as M34T is a controversial mutation associated with hearing loss (https://omim.org/entry/601544). Our expression construct initiating at M34, ran at the same size, strongly suggesting the truncated Cx26 form arises from this predicted initiation codon. The M34T deafness mutation completely abolished the expression of the truncated 23 k form. This suggests specific disease mutations may affect the translation of truncated isoforms.

Cx31.1-myc also displayed two strong bands (around 26–32 k), plus another band around 21 k (Fig. 1F). ΔM1 led to a loss of the slowest migrating 32 k band (presumably Cx31.1). The 26 k band was maintained and the 21 k band increased. A 20.5 k product was predicted *in silico* (bp 277), but the intensely expressed 26 k protein was not predicted. However, analyzing the Cx31.1 sequence (derived from HEK293T cells), we identified a reported single nucleotide polymorphism, Rs375424906, located at bp 97, which changes the GTG triplet to an ATG (M33) initiation site (predicted 25 k). Indeed, an M33 construct (initiating at bp 97) translated a protein of the same size. This poses the idea that specific single nucleotide polymorphisms can affect the finetuning of gene function by regulating the translation of truncated isoforms. Finally, to confirm the 21 k band likely initiated at M93 (bp 277–9), we transfected cells with a construct starting at M93, which yielded a protein the same size as the observed truncated isoform.

All the other connexins analyzed (Cx30, 31, 32, 40, 46) showed some evidence of potential truncated isoforms (with significant variations in the intensity and number), as summarized in Figure 1G–I. Notably, the ΔM1 mutation caused the loss of the major band predicted to be the FL proteins and maintained or increased (apart from Cx31), or further uncovered, additional truncated isoforms. This suggests that these isoforms are not a result of protein cleavage. However, several of these isoforms may stem from overexpression artefacts, and whether they occur *in vivo* remains to be determined. The *in silico* prediction concurred with the size of some but not all of the observed truncated isoforms and only served as a tool for orientation.

Alternative translation initiation, including through leaky ribosomal scanning, is emerging as a widespread mechanism driving protein diversity.^5^ Altogether, our data indicates that truncated isoforms may be translated throughout the connexin family, the extent of which can be influenced by single nucleotide polymorphisms and disease mutations. These are preliminary observations, but we urge the field to explore and expand upon these concepts in both health and disease, following the progress made with Cx43 over the last decade.

## CRediT authorship contribution statement

**María José Muñoz:** Conceptualization, Formal analysis, Investigation, Methodology, Writing – review & editing. **Alejandro Cabrera-Andrade:** Formal analysis, Investigation, Methodology, Writing – review & editing. **Ane Gutierrez-Aguirregabiria:** Investigation, Writing – review & editing. **Ipek Ilgin Gönenc:** Investigation, Writing – review & editing. **Santiago Ramón y Cajal:** Funding acquisition, Project administration, Resources. **Trond Aasen:** Conceptualization, Data curation, Formal analysis, Funding acquisition, Investigation, Methodology, Project administration, Resources, Supervision, Writing – original draft, Writing – review & editing.

## Funding

Trond Aasen acknowledges funding from 10.13039/501100004587Instituto de Salud Carlos III, grant PI21/00470 co-financed by the 10.13039/501100008530European Regional Development Fund.

## Conflict of interests

The authors declared no conflict of interests.

## References

[1] Smyth J.W., Shaw R.M. (2013). Autoregulation of connexin43 gap junction formation by internally translated isoforms. Cell Rep.

[2] Salat-Canela C., Sesé M., Peula C., Cajal S.R.Y., Aasen T. (2014). Internal translation of the connexin 43 transcript. Cell Commun Signal.

[3] Martins-Marques T., Ribeiro-Rodrigues T., Batista-Almeida D., Aasen T., Kwak B.R., Girao H. (2019). Biological functions of Connexin43 beyond intercellular communication. Trends Cell Biol.

[4] Chang T.H., Huang H.Y., Hsu J.B., Weng S.L., Horng J.T., Huang H.D. (2013). An enhanced computational platform for investigating the roles of regulatory RNA and for identifying functional RNA motifs. BMC Bioinformatics.

[5] Sriram A., Bohlen J., Teleman A.A. (2018). Translation acrobatics: How cancer cells exploit alternate modes of translational initiation. EMBO Rep.

