# Supplementary material for: Internal translation of truncated protein isoforms throughout the connexin gene family
Source: Genes Dis. 2025 Feb 25;13(2):101572. doi: 10.1016/j.gendis.2025.101572 (PMC12664632; doi:10.1016/j.gendis.2025.101572)
Supplement: Multimedia component 1 [file mmc1.docx]

**Table S1. In silico prediction of AUG translation initiation sites.** We used the software ^1^ (<https://atgpr.dbcls.jp/> ) to predict the 5 most likely synthesized proteins ^1^. We used the CDS including the upstream 6bp Kozak sequence to reflect the experimental conditions. The reliability score in brackets reflects the predicted score when analyzing the entire mRNA sequence (uORFs are not indicated).

| **Connexin**  ***GENE*** | **AUG No.** | **Reliability** | **Kozak** | **ORF Length**  **(aa)** | **Start (bp of CDS)** | **Stop (bp)** | **Estimated kD size** | **Main ORF?** |
| --- | --- | --- | --- | --- | --- | --- | --- | --- |
| **Cx43**  ***GJA1*** | **1** | **0.97 (0.62)** | **AxxATGG** | **382** | **1** | **1145** | **43,00** | **Y** |
|  | 15 | 0.16 | TxxATGc | 170 | 637 | 1145 | 18,53 | Y |
|  | 11 | 0.15 | GxxATGc | 258 | 373 | 1145 | 28,95 | Y |
|  | 20 | 0.14 | CxxATGt | 102 | 841 | 1145 | 11,26 | Y |
|  | 18 | 0.13 | AxxATGc | 82 | 794 | 1039 | 9,01 | N |
| **Cx46**  ***GJA3*** | **1** | **0.97 (0.85)** | **GxxATGG** | **435** | **1** | **1305** | **47,41** | **Y** |
|  | 5 | 0.43 | CxxATGg | 295 | 412 | 1305 | 31,20 | Y |
|  | 4 | 0.32 | CxxATGg | 334 | 304 | 1305 | 35,89 | Y |
|  | 9 | 0.28 | CxxATGa | 113 | 967 | 1305 | 11,33 | Y |
|  | 7 | 0.26 | AxxATGc | 217 | 655 | 1305 | 22,22 | Y |
| **Cx37**  ***GJA4*** | **1** | **0.97 (0.90)** | **GxxATGG** | **333** | **1** | **999** | **37,41** | **Y** |
|  | 9 | 0.30 | AxxATGg | 153 | 541 | 999 | 16,90 | Y |
|  | 4 | 0.22 | CxxATGg | 200 | 400 | 999 | 22,12 | Y |
|  | 6 | 0.21 | CxxATGg | 182 | 454 | 999 | 20,18 | Y |
|  | 11 | 0.16 | GxxATGa | 94 | 718 | 999 | 10,20 | Y |
| **Cx40**  ***GJA5*** | **1** | **0.69 (0.67)** | **AxxATGG** | **358** | **1** | **1074** | **40,38** | **Y** |
|  | 3 | 0.34 | GXXATGa | 111 | 140 | 472 | 11.93 | N |
|  | 7 | 0.31 | CxxATGc | 257 | 304 | 1074 | 28,81 | Y |
|  | 15 | 0.29 | AXXATGG | 86 | 818 | X | X | N |
|  | 10 | 0.27 | AXXATGG | 196 | 487 | X | X | N |
| **Cx32**  ***GJB1*** | **1** | **0.97 (0.57)** | **AxxATGa** | **283** | **1** | **849** | **32,02** | **Y** |
|  | 7 | 0.46 | GxxATCc | 191 | 277 | 849 | 21,74 | Y |
|  | 14 | 0.44 | GxxATGG | 122 | 484 | 849 | 13,44 | Y |
|  | 15 | 0.34 | GxxATGG | 90 | 580 | 849 | 13,44 | Y |
|  | 3 | 0.26 | AxxATGg | 250 | 100 | 849 | 28,22 | Y |
| **Cx26**  ***GJB2*** | **1** | **0.58 (0.69)** | **AxxATGG** | **226** | **1** | **678** | **26,21** | **Y** |
|  | 2 | 0.25 | AxxATGa | 193 | 100 | 678 | 22,48 | Y |
|  | 5 | 0.25 | GxxATGc | 134 | 277 | 678 | 15,85 | Y |
|  | 7 | 0.10 | TxxATGt | 76 | 451 | 678 | 8,73 | Y |
|  | 4 | 0.07 | GXXATGG | 24 | 228 | 299 | X | N |
| **Cx31**  ***GJB3*** | **1** | **0.60 (0.92)** | **GxxATGG** | **270** | **1** | **810** | **30,81** | **Y** |
|  | 5 | 0.33 | AxxATGc | 113 | 472 | 810 | 12,23 | Y |
|  | 2 | 0.22 | GXXATGa | 221 | 137 | 799 | X | N |
|  | 4 | 0.12 | GXXATGG | 113 | 461 | 799 | X | N |
|  | 6 | 0.10 | tXXATGG | 80 | 571 | 810 | X | N |
| **Cx31.1**  ***GJB5*** | **1** | **0.97 (0.77)** | **AxxATGa** | **273** | **1** | **819** | **31,08** | **Y** |
|  | 5 | 0.33 | GXXATGc | 161 | 255 | 737 | X | N |
|  | 6 | 0.16 | GxxATGc | 181 | 277 | 819 | 20,47 | Y |
|  | 11 | 0.15 | tXXATGG | 84 | 568 | 819 | 9.19 | Y |
|  | 10 | 0.13 | tXXATGt | 78 | 504 | 737 | X | N |
| **Cx30**  ***GJB6*** | **1** | **0.97 (0.59)** | **GxxATGG** | **261** | **1** | **783** | **30,38** | **Y** |
|  | 2 | 0.21 | GxxATGa | 228 | 100 | 783 | 26,64 | Y |
|  | 6 | 0.16 | GxxATGc | 169 | 277 | 783 | 19,98 | Y |
|  | 9 | 0.10 | txxATGt | 111 | 451 | 783 | 12,75 | Y |
|  | 15 | 0.05 | AXXATGc | 2 | 701 | 706 | X | N |
| **Cx45**  ***GJC1*** | **1** | **0.97 (0.40)** | **AxxATGa** | **396** | **1** | **1188** | **45,46** | **Y** |
|  | 22 | 0.24 | AxxATGt | 162 | 703 | 1188 | 18,05 | Y |
|  | 9 | 0.15 | AxxATGg | 295 | 304 | 1188 | 33,88 | Y |
|  | 24 | 0.14 | GxxATGc | 147 | 748 | 1188 | 16,34 | Y |
|  | 25 | 0.13 | cXXATGc | 91 | 917 | X | X | N |

**Supplementary Methods**

**Cloning**

All connexins were cloned from DNA extracted from the human HEK-293T cell line using the Phenol-Chloroform protocol. The purified PCR fragment was cloned into the pJET vector using the CloneJET PCR Cloning Kit (Thermo Scientific) before being transferred to the pLPCX retroviral vector where EGFP had been replaced by the myc tag sequence (EQKLISEEDL) via the XhoI / Bsu15I restriction sites. All plasmids were verified by sequencing.

**Cell Culture and transfections**

As previously described ^2^. Briefly All the cell lines were tested for Mycoplasma and cultured in DMEM (Dulbecco’s modified Eagle’s medium 4,5 g/L glucose (GIBCO, Thermo Fisher Scientific), supplemented with 10% fetal bovine serum (FBS) (Labclinics, Barcelona, Spain), 100 U/mL penicillin, 1000 µg/mL streptomycin (GIBCO, Thermo Fisher Scientific). Cells were maintained at 37°C and 5% CO2 in a humidified incubator. The cells were detached using TrypLeTM Express (GIBCO, Thermo Fisher Scientific). Cells were transfected with Polyethylenimine (PEI) used a 1 mg/mL stock solutyion (Plysciences) following standard transfection procedures.

**Western Blot Analysis**

As previously described ^2^. Briefly: Cells were washed twice with in PBS and then lysed with RIPA (Radioimmunoprecipitation) lysis buffer (Santa Cruz, Biotechnology) in presence of the protease (1:200) and phosphatase (1:100) cocktail inhibitors Calbiochem® (Merck). All the procedure was carried out on ice. The cellular lysate was collected in a 1.5 mL eppendorf tube, incubated 30 minutes on ice and mixed using the vortex every 10 minutes. To separate the cellular debris from the proteins the lysate was centrifuged at 15,000 x g during 20 minutes at 4°C. The pellet was discarded, and the supernatant was transferred to a clean new 1.5 mL eppendorf tube. From this protein isolation we determine the protein concentration obtained. The protein samples were prepared at the same concentration diluting them with the corresponding amount of RIPA lysis buffer and adding the loading buffer 1x (Laemli buffer 1x: 0,0626 M Tris-HCl pH 6,8; 2% SDS; 0,01% bromophenol blue; 10% glycerol). The proteins were loaded in Tris-glycine sodium dodecyl sulfate (SDS)-Polyacrylamide gels (SDS-PAGE) consisting of standard 5% stacking gel and 12% resolving gel. Proteins were denatured at 96°C for 5 minutes and 30 μg of each sample was loaded. Molecular weight marker: Precision Plus Protein Standards dual color (Bio Rad). Proteins were transferred to a PVDF (Polyvinylidene difluoride) membrane (Thermo Fisher) at 400 mA for 1 hour and 30 minutes. Membranes were blocked with 5% skimmed milk (DifcoTM) in T-TBS (Tris-HCl 50 mM pH 7,4; NaCl 150 mM and 0,1% Tween-20) for 30 minutes. After blocking, each membrane was incubated with the corresponding primary antibody in 5% skimmed milk in T-TBS (or 5% BSA (Bovine serum albumin) in T-BST) for 1 hour at room temperature followed by the incubation with the secondary antibody prepared in 5% skimmed milk in T-TBS. Immunoblotting was performed with rabbit anti-Cx43 (#C6219 Sigma Aldrich, 1:10000), Myc-Tag 9B11 Mouse mAb (#2276 Cell Signaling, 1:1000).

**Supplementary references:**

1. Salamov AA, Nishikawa T, Swindells MB. Assessing protein coding region integrity in cDNA sequencing projects. Bioinformatics. 1998;14(5):384-390. doi:10.1093/bioinformatics/14.5.384

2. Tishchenko A, Azorín DD, Vidal-Brime L, et al. Cx43 and Associated Cell Signaling Pathways Regulate Tunneling Nanotubes in Breast Cancer Cells. Cancers (Basel). 2020;12(10):2798. doi:10.3390/cancers12102798
